# A Quality Improvement Project of Acute Red Eye Consultations in Primary Care: Improving the Identification of Red Flags

**DOI:** 10.7759/cureus.50344

**Published:** 2023-12-11

**Authors:** Amrit Hayre

**Affiliations:** 1 Foundation Programme, University College London Hospitals NHS Foundation Trust, London, GBR

**Keywords:** conjuntivitis, ophthalmology consultations, quality improvement, ophthalmology, red flag symptoms, acute red eye

## Abstract

Aim

The acute red eye or conjunctival injection is the most common ophthalmic presentation in primary care. The aim of this audit was to improve red eye consultations by increasing the number of red flag features documented in the history of patients.

Method

The National Institute of Clinical Excellence (NICE) Clinical Knowledge Summary (CKS) outlines red flag features, which should be documented for an acute red eye consultation. Two interventions were implemented to attempt to improve consultations. The first involved creating a template in the Egton Medical Information Systems. The second involved training doctors in the recognition of red flag features and identifying sight-threatening conditions. Red eye consultations and red flag features documented were recorded in the two months before and after the interventions.

Results

All documentation of red flag features assessed in this project increased post intervention. However, lateralisation, visual changes, and eye pain were commonly asked prior to the intervention and did not show a statistically significant difference. They showed an increase from 90% (19/21) to 100% (19/19), 71% (15/21) to 84% (16/19), and 67% (14/21) to 79% (15/19), respectively. After the interventions, significant increases in asking about headaches (14% (3/21) to 74% (14/19), pupil changes (5% (1/19) to 58% (11/19)), and method of injury (high velocity 10% (2/21) to 84% (16/19), foreign body 14% (3/21) to 84% (16/19), chemical 10% (2/21) to 84% (16/19) were observed (p<0.05). Photophobia inquiries also significantly increased (14% (3/19) to 79% (15/19), P<0.05).

Conclusion

The number of red flag features identified and documented for acute red eye consultations increased with the introduction of an online template and a teaching session.

## Introduction

The most common ophthalmology presentation in primary care is acute red eye [1]. The overwhelming majority are diagnosed as conjunctivitis but sight-threatening conditions can also present with this symptom [2]. Therefore, the timely identification and appropriate referral of patients with red-flag symptoms is necessary to prevent serious adverse outcomes. An analysis of patients with acute red eye found that those with red flag features were more likely to have a serious diagnosis, such as acute angle-closure glaucoma or scleritis than those without red flag features [3]. Similarly, a study of patients with acute red eye demonstrated that the presence of red flag features was significantly associated with a higher likelihood of referral to an ophthalmologist and a correct diagnosis [4]. Despite this, one study showed that in general practitioner (GP) practice/surgeries, only 36% of patients with red flags features of red eye were appropriately referred to ophthalmology [3]. The Royal College of General Practitioners (RCGP) highlighted that half of instances of sight loss could be prevented by improving eye care and detecting issues early on [5]. The National Health Service (NHS) acknowledges a nationwide problem concerning the timely follow-up of glaucoma patients. Studies indicate that approximately 22 patients experience severe or permanent vision loss each month due to these delays [6].

The list of red flag features to be screened for by the National Institute of Clinical Excellence (NICE) Clinical Knowledge Summary (CKS) has been revised multiple times since its conception. Initially, only five screening questions were suggested, which included questions relating to pain, photophobia, vision changes, whether the condition is unilateral or bilateral and foreign body or penetrating eye injury [7]. The latest guidance from NICE CKS lists the following symptoms as red flags for patients who present with acute red eye: Reduced visual acuity, moderate to severe pain, headache, photophobia, unilateral, a high-velocity injury, or injuries involving glass, foreign body or penetrating injury or trauma, serious chemical eye injury, and contact lens use [8]. 

NICE CKS also mention red flag signs. As this quality improvement project is focussing on consultations in a GP practice/surgery with limited ophthalmology examining equipment such as dilating eye drops, slit lamp, etc., and most patient appointments being via telephone, red flag signs have not been audited. The aim of this audit was to improve red eye consultations by ensuring red flag features were asked with an overall goal to timely refer appropriate patients to specialist services.

## Materials and methods

Regents Park Practice uses Egton Medical Information Systems (EMIS) (EMIS Health, Leeds, United Kingdom) as their electronic paper record. Within EMIS, all patient consultations coded with ‘acute red eye’ over a two-month period, between April and May 2023, were selected. Acute red eye was defined as a new symptom that has presented within the past two weeks. There was no exclusion criterion for age and sex, etc.; however, all consultations analysed were completed by physicians. All patient details were removed and anonymised. The number of red flag features documented, positive or negative, were recorded in Excel (Microsoft Corporation, Redmond, Washington, United States). Positive findings were recorded as 1, and negative findings as 0. The number of positive findings was divided by the n number and multiplied by 100 to give the mean percentage. It is important to note that pain was assessed by asking the patient to score their pain between 0-10. A pain score of 7 or greater was considered moderate/severe.

**Figure 1:**
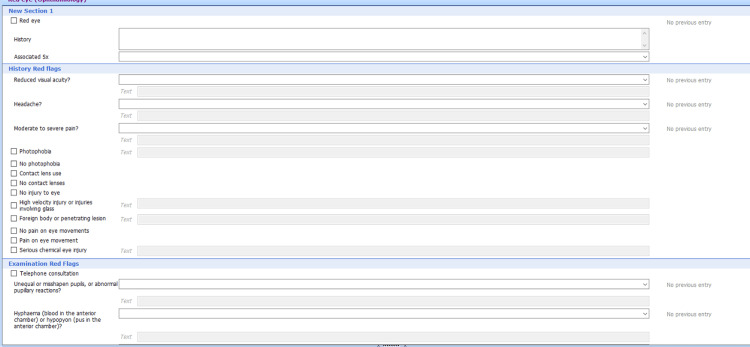
An EMIS template created for consultations for patients presenting with acute red eye. Red flag features are displayed and can be ticked if present or not present EMIS: Egton Medical Information Systems (EMIS Health, Leeds, United Kingdom)

**Figure 2:**
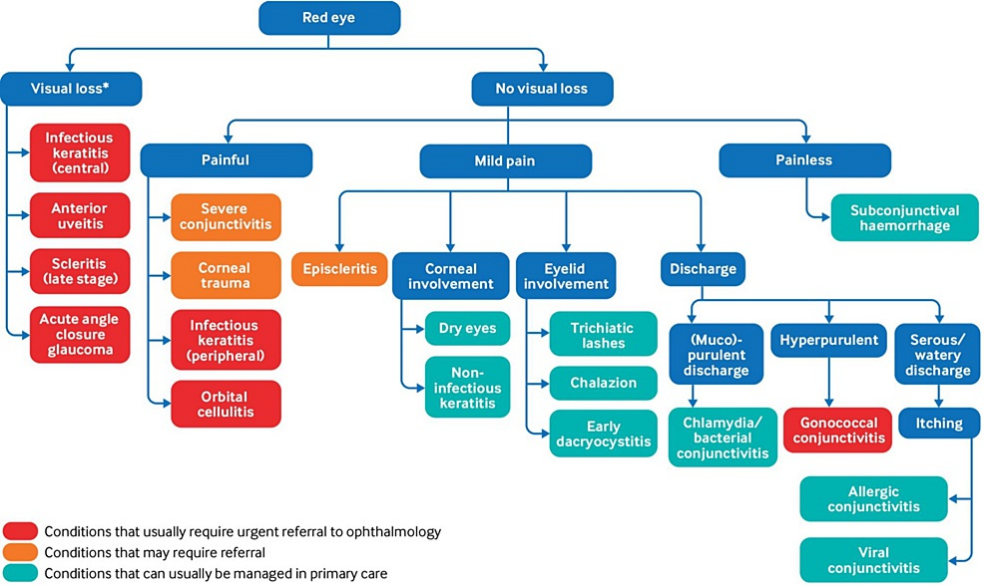
Red eye differential diagnosis algorithm used for teaching acute red eye consultations in a GP Practice GP: general practitioner Image Source: Ho et al., 2021 [9]; Open Access, CC BY-NC 4.0 license

For part I of the intervention, a template was created in EMIS (Figure 1) for patients presenting with ‘acute red eye’. The template consisted of tick boxes of the NICE CKS red flag features, stating whether the patient presented with or without the feature. Part II of the intervention was to teach the doctors at the GP practice how to use the template. The session also included which red flag features to look out for, how to extract that information from the patient, sight-threatening conditions that present with red eyes, and which patients to refer to secondary care. The teaching material used during the session is displayed in Figure 2. 

All consultations coded with 'red eye' were re-audited two months later, between June and July 2023, using the same methodology as in cycle 1. The number of red flag features documented, positive or negative, was recorded once again. An unpaired T-test was used to analyse all parametric data to give a p-value for significant changes. 

## Results

During consultations with patients presenting with an acute red eye, the most asked question was lateralisation of their symptoms, which was asked 19/21 times at baseline and 19/19 times after the interventions (Table 1). This was not a statistically significant increase (p=0.65). Similarly, pain and vision changes were commonly asked by GPs prior to the intervention at a rate of 14/21 and 15/21 consultations, respectively (Table 1). Post intervention, these symptoms were asked more often at a rate of 15/19 and 16/19, respectively, yet did not show a statistical difference (pain, p=0.43; vision changes, p=0.38) (Table 1).

**Table 1:** Red flag features documented (positive or negative) in pre-intervention and post-intervention consultations

Red flag symptoms	Pre-intervention (n=21)	Post-intervention (n=19)	T-test
Lateralisation	19	19	P = 0.6452
Pain	14	15	P = 0.4258
Headache	3	14	P = 0.0001
Vision changes	15	16	P = 0.3769
Pupil changes	1	11	P = 0.0001
High-velocity injury	2	16	P < 0.0001
Foreign body injury	3	16	P < 0.0001
Chemical injury	2	16	P < 0.0001
Contact lens wearer	1	15	P < 0.0001
Photophobia	3	15	P < 0.0001

Asking about headaches significantly increased from 3/21 consultations to 14/19 consultations (p<0.05) (Table 1). Similarly, asking about pupil changes increased from 1/21 consultations to 11/19 consultations, a significant increase from baseline (p<0.05) (Table 1).

**Figure 3:**
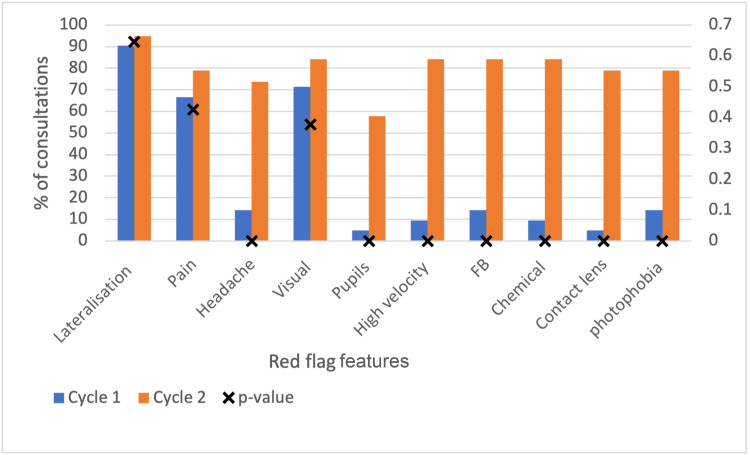
Consultations in which red flag features were asked of patients presenting with acute red eye.

The method of injury covers three red flag features: high velocity, foreign body, and chemical injury. All three had a significant increase (p<0.05) after the intervention (Table 1, Figure 3). High-velocity injuries went from being documented in 10% of consultations (2/21) to 84% of consultations (16/19), foreign body documentation increased from 14% (3/21) to 84% (16/19) and chemical injury documentation increased from 10% (2/21) to 84% (16/19) of consultations. Three patients out of 21 were asked if they had experienced photophobia in the first cycle, which increased to 15/19 after the intervention, in cycle 2. This was a statistically significant increase (p<0.05) (Table 1).

## Discussion

This audit highlighted that some of the more common questions related to red flag features were consistently included in most GP consultations for acute red eye cases, whereas other red flag features were almost completely omitted. The general feedback received from the doctors at the GP practice was a lack of understanding of less common ophthalmology conditions that required urgent referral. This feeling has been studied in the literature and is not unique to doctors at Regents Park Practice [10]. Furthermore, they were not confident in identifying and addressing all pertinent red flags. This is likely explained by the recent update of the national guidance by adding more detailed screening questions [7]. A quality improvement project by Teo showed that after the intervention of education with reinforced memory aids and improving the accessibility to eye examining equipment, documentation of four screening questions relating to pain, photophobia, vision changes, and lateralisation increased from 2.8% to 50% for acute red eye consultations [11]. The limitation of the 2014 audit was that all screening questions were not assessed. Looking at consultations in this audit, it is clear that GPs were confident in enquiring about the same red flag features assessed by Teo at baseline. However, after both interventions, it appears that GPs at Regents Park Practice were more confident in identifying all relevant red flags due to the increased number of documented red flags, both positive and negative. 

These results are not unique to Regent’s Park Practice as other primary care practices also showed that implementing teaching and visual reminders via posters showed an increase in the number of red flag feature questions asked for in acute red eye cases [12]. This highlights the lack of understanding of serious ophthalmology conditions in multiple primary care centres around the country, which can be improved with simple interventions. Although GPs are a good filter for ophthalmic referrals, providing them with specialist teaching and systematic aids has been shown to aid more appropriate referrals in other specialities [13].

The implementation of this quality improvement project is sustainable as once the proforma has been made it can be repeatedly used and implemented in all primary care practices with EMIS. With trainees and locum doctors making up a proportion of primary care doctors, there tends to be a high turnover in staff; hence, teaching in part II of the intervention will need repeating. To mitigate this not happening regularly or effectively, guidelines for urgent ophthalmology referral were attached to the proforma. This project's additional advantage lies in the fact that all the interventions could be implemented at no cost, enhancing its reproducibility in other centres. The project could also be extended to include other healthcare professionals who refer to ophthalmologists, such as optometrists. Studies indicate that optometrists refer more patients to acute ophthalmology centres but with a higher false positive rate compared to GPs [14,15].

Even though statistically significant results were obtained, a clear limitation of this study is the low number of consultations assessed. In future, this project should be reproduced on a regional scale. To progress this project, it would be useful to see how this change affected urgent ophthalmology referrals, specifically details on the number of referrals and the number of appropriate referrals. Reduction in inappropriate referrals would free up time and resources in eye casualty. However, it must be noted that it becomes challenging to assess the appropriateness and outcome of referrals when a crucial patient subgroup is overlooked, specifically those with similar symptoms and conditions who were not referred. NICE also recommends screening for red flag signs in patients presenting with an acute red eye; hence, further audits should focus on recording improvements in this domain. This could be achieved by giving clinicians better access to ophthalmology examination equipment such as a slit lamp, dilating eye drops, fluorescein drops, or simply education on how to spot the pertinent signs. 

## Conclusions

The audit underscores the inconsistency in addressing red flag features during GP consultations for acute red eye cases, revealing a particular lack of attention to less common ophthalmology symptoms. With the introduction of an online template and a teaching session, the number of red flags identified and documented for acute red eye consultations increased at Regents Park Primary Care Practice. These findings corroborate with the current literature that education and systems can be introduced to improve the quality of acute red eye consultations in primary care. 
